# High-resolution gridded population datasets for Latin America and the Caribbean using official statistics

**DOI:** 10.1038/s41597-023-02305-w

**Published:** 2023-07-07

**Authors:** Tom McKeen, Maksym Bondarenko, David Kerr, Thomas Esch, Mattia Marconcini, Daniela Palacios-Lopez, Julian Zeidler, R. Catalina Valle, Sabrina Juran, Andrew J. Tatem, Alessandro Sorichetta

**Affiliations:** 1 WorldPop, School of Geography and Environmental Science, University of Southampton, Southampton, UK; 2 German Aerospace Centre (DLR), Wessling, Germany; 3 United Nations Population Fund (UNFPA), Regional Office for Latin America and the Caribbean, Panama, Panama; 4 Dipartimento di Scienze della Terra “A. Desio”, Università degli Studi di Milano, Milano, Italy

**Keywords:** Sustainability, Geography, Natural hazards

## Abstract

“Leaving no one behind” is the fundamental objective of the 2030 Agenda for Sustainable Development. Latin America and the Caribbean is marked by social inequalities, whilst its total population is projected to increase to almost 760 million by 2050. In this context, contemporary and spatially detailed datasets that accurately capture the distribution of residential population are critical to appropriately inform and support environmental, health, and developmental applications at subnational levels. Existing datasets are under-utilised by governments due to the non-alignment with their own statistics. Therefore, official statistics at the finest level of administrative units available have been implemented to construct an open-access repository of high-resolution gridded population datasets for 40 countries in Latin American and the Caribbean. These datasets are detailed here, alongside the ‘top-down’ approach and methods to generate and validate them. Population distribution datasets for each country were created at a resolution of 3 arc-seconds (approximately 100 m at the equator), and are all available from the WorldPop Data Repository.

## Background & Summary

The United Nations (UN) projects that the global human population will grow by 2 billion between 2019 and 2050^1^. Specifically, Latin America and the Caribbean has a total population of approximately 658 million, and is expected to increase by approximately 90 million by 2050^1^.

The region has made important strides against infant and maternal mortality, communicable disease transmission, and incidence of noncommunicable disease in the last 10 years^2^, largely due to economic development, and the improved capacity and flexibility of healthcare systems^3,4^. However, the challenge to overcome inequalities of health outcomes derived from the intersection of determinants including socio-economic status, gender, and ethnicity at subnational levels is identified as a key step to universal health access, a key target of the UN Sustainable Development Goals (SDGs)^1,2^. Moreover, geographic access is a principal determinant of healthcare access, and is crucial to identifying inequities in subnational health status and access to healthcare^5,6^.

According to the UN Office for the Coordination of Humanitarian Affairs^7^, Latin America and the Caribbean is the second most disaster-prone region in the world, with 152 million people impacted by 1,205 disasters between 2000 and 2019. Hydrometeorological phenomena including flooding, storm surges, and hurricanes are the most common and destructive hazards in the region^7^, comprising 60% of all reported disasters during 2010–2016, at an estimated cost of US$278 million dollars^2^. Climate change operates as a ‘risk magnifier’, increasing the volatility and frequency of hazard events, which disproportionately impacts the populations of low- and lower middle-income countries^8,9^. Small-island territories and major coastal settlements are particularly threatened by sea-level rise^8^, with an estimated 30 million people living in low-lying areas (i.e. within the first 10 m of elevation) in the region^10^. Moreover, the region is exposed to significant seismic and volcanic activity^11^, due to its location along the ‘Ring of Fire’, a belt following the edge of the Pacific Ocean encountering 80% of the world’s volcanic and seismic events^12^. Between 2000 and 2019, 75 earthquakes occurred in the region, resulting in 226,000 deaths at a total damages cost of US$54 billion^7^.

Consequently, efforts towards a fuller and clearer understanding of the spatial distribution of population is crucial to a whole swathe of developmental goals. Amongst natural and man-made disaster scenarios there is a demand for high-resolution population estimates to support the accurate assessment of the scale of an event and the required relief^13–16^. Since such hazard events are highly unlikely to impact areas conforming to administrative units, detailed WorldPop gridded data is already regularly used to more precisely assess the size and characteristics of potentially affected population, typically age and sex^17,18^. Moreover, accurate population estimations are fundamental to nearly all public health intervention and planning efforts^19,20^. Regularly updated estimates facilitate an enhanced understanding of population size and distribution, improving the efficiency and effectiveness of targeted vaccination planning and delivery programmes^21^.

Therefore, significant work has been undertaken since the early 1990s to develop high-resolution gridded population datasets at global or continental scales^22^. Advancements in the spatial resolution and availability of geospatial data, statistical analysis approaches, and processing power have enabled the generation of more accurate datasets that describe changes in human population scale, composition, and distribution over time^23^. These advancements have facilitated the development of a wide range of openly available, large-scale gridded population datasets^22,24–32^. However, these datasets have been of limited value to governments due to the lack of alignment with their own population figures. Therefore, seeking to overcome this limitation and encourage the uptake of gridded population data, this project represents the first endeavour to use official population figures and boundaries to create gridded population data across an entire continental region.

WorldPop is an interdisciplinary applied research program that develops peer-reviewed research and methods for the construction of open and high-resolution geospatial data on population distribution, demographics, and dynamics. Within this framework, an open-access repository of high-resolution gridded population datasets for the Latin America and the Caribbean region has been generated, using official, finest-available population census-based figures and projections (Table 1) and national boundaries provided by National Statistic Offices (NSOs) from the region, alongside a suite of ancillary geospatial datasets relating to human population, including high-resolution settlement data. Using a Random Forest (RF) dasymetric modelling approach^33^, population count data and ancillary geospatial datasets for 40 countries (Tables 1, 2) were gathered, prepared, and processed to create gridded population datasets with a spatial resolution of 3 arc-seconds (approximately 100 m at the equator).

**Table 1:** Summary information of population count data and administrative boundary datasets used to produce the gridded population datasets.

ISO	Area (km^2^)	Total population	No. of units	Unit level	ASR	Modelled with	Year	Base-census year
ABW	177	112,683	55	2	0.24		2020 ^P^	2010
AIA	76	13,572	9	0	0.97	Grouped Islands*	2011^C^	2011
ARG	2,779,164	45,808,456	525	2	3.18		2021 ^P^	2010
ATG	414	84,816	8	1	2.54	Grouped Islands*	2019^P^	2017
BHS	11,859	391,476	32	1	3.40	Grouped Islands*	2019^P^	2010
BLZ	21,764	322,454	6	1	24.59	GTM	2020 ^P^	2010
BMU	50	63,779	11	2	0.64	Grouped Islands*	2020 ^P^	2016
BOL	1,081,700	11,841,955	9	1	115.56	PER, PRY	2021 ^P^	2012
BRA	8,478,053	211,755,692	5,570	2	0.52		2020 ^P^	2010
BRB	431	226,193	11	1	1.89	Grouped Islands*	2010^C^	2010
CHL	749,230	19,678,363	346	3	3.52		2021 ^P^	2017
COL	1,136,979	50,372,424	1,122	2	0.95		2020 ^P^	2018
CRI	51,061	5,163,021	478	3	0.47		2021 ^P^	2011
CUB	109,272	11,193,470	168	2	1.97		2015^P^	2012
CUW	430	165,983	65	1	0.32	Grouped Islands*	2020 ^P^	2015
CYM	257	69,914	6	1	2.67	Grouped Islands*	2019^C^	2016
DMA	747	69,325	10	1	2.73	Grouped Islands*	2011^C^	2011
DOM	47,914	10,448,499	155	3	1.41		2020 ^P^	2015
ECU	254,784	17,510,643	25	1	20.19	COL	2020 ^P^	2010
GRD	347	114,000	7	1	2.66	Grouped Islands*	2020 ^P^	2011
GTM	108,193	17,109,746	340	2	0.97		2021 ^P^	2018
GUY	210,000	756,237	10	1	45.83	SUR	2021 ^P^	2012
HND	111,460	9,302,282	298	2	1.12		2020 ^P^	2013
JAM	10,948	2,697,983	14	1	7.47	DOM	2019^P^	2011
KNA	261	46,325	14	1	1.15	Grouped Islands*	2020 ^P^	2011
LCA	594	178,696	10	1	2.44	Grouped Islands*	2018^P^	2011
MEX	1,948,457	128,972,439	2,457	2	0.57		2021 ^P^	2010
MSR	99	4,566	39	EA	0.26	Grouped Islands*	2021 ^P^	2011
NIC	127,982	6,595,672	153	2	2.34		2020 ^P^	2012
PAN	74,153	4,337,406	13	1	20.95	HND, SLV	2021 ^P^	2010
PER	1,286,915	29,381,884	1,873	3	0.61		2021 ^P^	2017
PRY	399,572	7,252,669	250	2	2.53		2020 ^P^	2012
SLV	20,382	6,825,935	262	2	0.54		2021 ^P^	2007
SUR	146,242	590,100	62	2	6.17		2018^P^	2011
TCA	943	31,458	6	1	5.12	Grouped Islands*	2021 ^P^	2012
TTO	5,129	1,328,022	15	1	4.77	Grouped Islands*	2020 ^P^	2005
URY	177,398	3,530,912	19	1	22.17	COL, PRY	2020 ^P^	2011
VCT	383	110,696	13	2	1.51	Grouped Islands*	2018^P^	2012
VEN	912,709	32,605,423	25	1	38.21	PRY	2020 ^P^	2011
VGB	150	32,670	25	0	0.49	Grouped Islands*	2017^P^	2010

Each country is identified by its ISO-3 country code (https://www.worlddata.info/countrycodes.php). The average spatial resolution (ASR) was calculated as the square root of each country’s surface area divided by the number of administrative units, and represents the effective resolution of the latter (i.e., the cell size of administrative units if all units were square of equal size)^22^. Countries with fewer than 25 administrative units were modelled with additional countries, selected based on similar characteristics; ‘Grouped Islands’ refers to a set of countries that were modelled simultaneously, including: BHS, TCA, CYM, VGB, KNA, ATG, MSR, DMA, LCA, BRB, GRD, TTO, BMU, VCT, AIA, MSR, VGB. Superscripts ‘C’ or ‘P’ in the 8^th^ column, indicate whether population statistics were obtained from either official census or census-based projections, respectively.

**Table 2:** Summary information of the default datasets and the derived default covariates used for RF model fitting and prediction.

Default dataset	Default derived covariate	Temporal coverage	Type	Format	Resolution	Source
**SNPP-VIIRS**		**2020**	**Continuous**	**Raster**	**15 arc seconds**	**EOG**^52^
	Night-light intensity	2020	Continuous	Raster	3 arc seconds	
**HydroSHEDS (GRID: Void-filled DEM)**		**2006**	**Continuous**	**Raster**	**3 arc seconds**	**WWF**^53^
	Elevation	2006	Continuous	Raster	3 arc seconds	
	Slope	2006	Continuous	Raster	3 arc seconds	
**ESA-CCI Ocean/Inland/Land Waterbodies**		**2000**–**2012**	**Categorical**	**Raster**	**150 m**	**ESA-CCI**^58^
	Distance to inland water	2000–2012	Continuous	Raster	3 arc seconds	
	Ocean watermask	2000–2012	Categorical	Raster	3 arc seconds	
	Inland water/permanent ice mask	2000–2018	Categorical	Raster	3 arc seconds	
**C3S Global Land Cover**		**2018**	**Categorical**	**Raster**	**300 m**	**ESA-CCI**^59^
	Distance to class #	2018	Continuous	Raster	3 arc seconds	
	Inland water/permanent ice mask	2018	Categorical	Raster	3 arc seconds	
**WSF3D Building Area**		**2021**	**Continuous**	**Raster**	**90 m**	**Esch** ***et al***^68^*.*
	Building area footprint	2021	Categorical	Raster	3 arc seconds	
	Building area	2021	Continuous	Raster	3 arc seconds	
	Distance to urban area	2021	Continuous	Raster	3 arc seconds	
**WSF3D Building Height**		**2021**	**Continuous**	**Raster**	**450 m**	**Esch** ***et al***^68^*.*
	Building height	2021	Continuous	Raster	3 arc seconds	
	Building volume	2021	Continuous	Raster	3 arc seconds	
**Roads/road intersections features**		**2021**	**Categorical**	**Vector**	**/**	**Geofabrik**^63^; **BBBike**^64^
	Distance to road classes	2021	Continuous	Raster	3 arc seconds	
	Distance to intersection classes	2021	Continuous	Raster	3 arc seconds	
	Road length	2021	Continuous	Raster	3 arc seconds	
	Road density	2021	Continuous	Raster	3 arc seconds	
	Road intensity (4 bandwidths)	2021	Continuous	Raster	3 arc seconds	
**Distance to IUCN protected area edges**		**2017**	**Continuous**	**Raster**	**3 arc seconds**	**WorldPop**^66^
	Distance to wildlife protected areas	2017	Continuous	Raster	3 arc seconds	
**Distance to OSM major waterways**		**2016**	**Continuous**	**Raster**	**3 arc seconds**	**WorldPop**^66^
	Distance to waterways	2017	Continuous	Raster	3 arc seconds	
**Distance to open-water coastline per country**		**2020**	**Continuous**	**Raster**	**3 arc seconds**	**WorldPop**^66^
	Distance to coastline	2020	Continuous	Raster	3 arc seconds	
**Grid cell surface areas**		**2020**	**Continuous**	**Raster**	**3 arc seconds**	**WorldPop**^66^
	Pixel area	2000–2020	Continuous	Raster	3 arc seconds	
**Climate (Temp/Precipitation)**		**1970**–**2000**	**Continuous**	**Raster**		**Fick & Hijmans**^65^
	Temperature	1970–2000	Continuous	Raster	3 arc seconds	
	Precipitation	1970–2000	Continuous	Raster	3 arc seconds	

Continuous raster datasets were resampled for use as covariates, whilst both categorical and rasterized datasets were firstly resampled and then processed into *‘presence/absence*’ or ‘*distance to*’ raster covariates.

## Methods

The methodology used to construct this data product, similarly to previous WorldPop products for the region^28^, implements a top-down approach to population disaggregation via a RF dasymetric modelling approach^33^. However, there are two marked differences underlying the data product presented herein: i) the use of official, finest-available census-based population figures and projections (Table 1) and administrative boundaries, and ii) the addition of high-resolution World Settlement Footprint 3D (WSF3D) data to the suite of RF-fitting covariates.

### Random forest-based dasymetric population mapping approach

A RF algorithm was implemented to generate a gridded population density weighting layer at 3 arc-second resolution (approximately 100 m resolution at the equator); this prediction layer is then used to perform dasymetric disaggregation of population counts from administrative units into target grid cells at country level^33^. RF is a predictive, non-linear, and non-parametric ensemble learning approach that generates a large set of decision tree models and aggregates their predictions^34^. Decision trees are independently generated by bagging (i.e., by sampling the entire dataset with replacement)^35^, typically two thirds of samples are used to train the trees (known as the ‘*bagged*’ sample). Each node of each decision tree is split according to an iterative method in which, at each node, the optimal splitting method is used^34^. After all regression trees have been constructed, the outputs of all tree predictions are aggregated by calculating either their mode or average, contingent on whether the trees are utilised for classification or regression, to produce a final classification decision^36^. The remaining third of unsampled data, known as ‘*out-of-bag*’ (OOB), are used to perform the internal cross-validation technique to accurately estimate the prediction error of the RF model^34^; this is achieved by averaging all mean squared errors calculated using the OOB data. The RF approach is robust to overfitting^34^, and its predictive accuracy is not very sensitive to the three parameters to be specified for model fitting^36^, explicitly, (i) the number of observations in the terminal nodes of each tree, (ii) the number of trees in the forest, and (iii) the number of covariates to be randomly selected at each node.

The RF-based dasymetric population mapping approach developed by Stevens *et al*.^33^, has been used in this framework to produce gridded population distribution datasets for Latin American and Caribbean countries. This approach consists of using a RF algorithm to generate gridded population density estimates that are subsequently used, as a weighting layer, to dasymetrically disaggregate population counts from administrative units into grid cells^37^.

RF model fitting was undertaken by generating 500 trees, and assigning the number of observations in the terminal nodes equal to one. Following RF model fitting, population density was predicted using a reduced selection of covariates. For each target grid cell, the average of all decision tree predictions was designated to the cell as the estimated population density value. Where there were insufficient observations (i.e. insufficient administrative unit population counts) to fit a RF model for a given country, an additional country with similar characteristics was selected, and utilised to fit an appropriate RF model for predicting population density at the grid cell level^38^. Subsequently, in both scenarios, dasymetric disaggregation of the administrative unit-based population counts was undertaken using the population density weighting layer^37^, thereby generating two gridded population datasets of estimated number of people per grid cell.

All tasks described above were performed using the popRF package in R^39^. The popRF package functionalises the RF-informed dasymetric population modelling procedure^33^ within a single programming language framework, and is publicly available, open source, and environment agnostic^39^. This package has been parallelised where possible to achieve efficient prediction and geoprocessing over large extents, supporting functions that have applied utility beyond simply performing disaggregative population modelling^39^.

### Data collection

For every country listed in Table 1, population counts were paired with their corresponding administrative unit boundaries within a GIS interface. Official and best available subnational population census-based figures and projections, and at the finest administrative unit level possible, alongside matching official administrative unit boundaries were provided by NSOs of the region with support from the UNFPA and OCHA. These input data are technically assessed by the UNFPA and subject-matter country experts, and are adopted as common baseline population data for use in disaster preparedness and operational humanitarian response. Further summary information regarding the input population data, including base-census year, and corresponding administrative unit boundaries is provided in Table 1.

Human population density is known to be highly influenced and correlated with a variety of environmental and physical factors, each of which can be credibly associated with and influence the spatial distribution of population^23,30,40^. These factors are classified into two distinct categories; firstly, continuous variables such as topographic elevation and slope^41,42^, climate^43^, and intensity of night-time lights^44,45^. Secondly, categorical variables notably including land cover type^46,47^ and the presence or absence of settlements and urban areas^48^, roads^48^, waterbodies and waterways^49^, and protected areas^50^. Therefore, the 12 most up-to-date global raster and vector datasets available at the time of production, were identified, collected, and processed into a uniform set of default covariates used for model fitting and prediction (Table 2).

The spatial variation of factors related to population distribution, such as night-light intensity, was measured using nightly day/night band (DNB) low-light imaging data collected by the Visible Infrared Imaging Radiometer Suite (VIIRS) aboard the Suomi National Polar Partnership (SNPP) satellite^51,52^. HydroSHEDS data^53,54^, derived from NASA’s Shuttle Radar Topography Mission (SRTM) elevation data^55^, was used to generate elevation and slope covariates. Specifically, the 3-arc second, void-filled digital elevation model (DEM) product was implemented^56^.

A global dataset of inland and ocean water was acquired from the European Space Agency’s (ESA) Climate Change Initiative (CCI) land cover project at a spatial resolution of 150m^57,58^. The data was built within the ESA-CCI project framework for the 2000–2012 period and enabled the generation of inland and ocean water masks. Global gridded land cover (LC) data for 2018 was obtained via the Copernicus Climate Change Service (C3S), using Intermediate Climate Data Records (ICDR) produced by the ESA-CCI project^59–61^. This data was used to identify different land cover types, and generate distance to land cover class covariates, whilst the permanent ice land cover class was incorporated with the ESA-CCI waterbodies dataset to generate a global mask of inland water and permanent ice. This global watermask was used to identify areas of non-human habitation due to the presence of waterbodies. The final stage of production for all covariates masked pixels identified as containing water, setting pixels to ‘*No Data*’ within these areas.

OpenStreetMap^62^ vector datasets were extracted for road and road intersection features via two distinct data repositories Geofabrik^63^ and BBBike^64^, respectively. Temperature and precipitation data, representative of the 1970–2000 period, were downloaded from WorldClim, version 2.1 climate data for 1970–2000^65^. Moreover, a selection of pre-prepared covariates was extracted from the WorldPop open access gridded data archive to complete the set of covariates required for model fitting and prediction^66^. All data was available at 3 arc-second resolution, and had been already fully harmonised to support population distribution prediction applications^27,67^. These datasets included time-invariant covariates: distance to waterways, protected areas, and coastlines (Table 2).

The DLR’s World Settlement Footprint 3D (WSF3D) product was used to identify, quantify, and calculate distance to settlement in this research. The processing methodology of the WSF3D product is based on work presented by Esch *et al*.^68^. The WSF3D production approach is dependent on two predominant input datasets: (i) the 12 m spatial resolution TDX_DEM, and (ii) an updated version of the WSF imperviousness (WSF-Imp) dataset displaying the percent of impervious surface at ~10 m spatial resolution^69,70^ within the built-up area defined by the WSF2019 human settlement mask^71,72^. The DLR provided layers for each of the 40 countries specific to this research. A short description of each layer’s production process is denoted below^68,73^:

#### Building height (BH)

The ~450 m BH layer represents a spatial aggregation of the standard 90 m WSF3D BH layer, which was derived by measuring the height variations of vertical edges most likely related to building edges (BE) in the 12 m TDX-DEM within the settlement areas defined by the WSF-Imp layer. The height is reported in metres (m) in the final product.

#### Building area (BA)

The ~90 m BA layer is derived by firstly generating the Building Fraction (BF) layer, which measures percentage building coverage per ~90 m cell in a range of 0–100. This is produced by quantifying the built-up coverage at 12 m spatial resolution, derived from the joint analysis of the WSF-IMP, TDX-amplitude images (TDX-AMP), and BE. The BF is subsequently multiplied by the area of each ~90 m grid cell (~8100 m^2^ at the equator), thereby producing the BA. This area is reported in square metres (m^2^) in the final product.

### Data processing

The population count data for each country was manually cleaned, processed, and harmonised to match to its corresponding official vector administrative unit dataset. The administrative and population count data was recoded, adding a ‘*GID*’ primary key field through which each row in the two datasets could be joined.

For each country (Table 1), the vector dataset representing its administrative units, used to match to the population counts, was cleaned and projected using the WGS 1984 geographic coordinate system; this system was selected to ensure uniformity across all generated covariate datasets. These datasets were then buffered by 100 km extent and rasterized at a resolution of 3 arc-seconds (approximately 100 m at the equator). These measures were taken to: (i) obtain a raster dataset of the study area to register and ensure uniformity across all raster covariates, (ii) produce a set of raster ‘distance to’ covariates that were unaffected by artificial boundary effects throughout spatial processing^74^, and (iii) conduct spatial processing on a buffered country-level basis, rather than on a global scale, to save processing time where necessary.

Default input covariates for the RF model were derived as follows. In most cases, raw datasets required specific cleaning and conversion methods to ensure format accessibility for further spatial processing. All raster variables representing continuous values (Table 2), were projected to WGS 1984 datum, resampled to 3 arc-second resolution, and matched to the rasterized study area. ‘*No Data*’ grid cells overlapping the rasterized buffered study area extent were filled with values of the nearest neighbour (using the Nibble tool available in ArcGIS 2.7.1). Finally, each covariate variable was extracted to the rasterized study area extent, maintaining uniformity of spatial extent, and resolution. All vector and raster datasets representing categorical variables were projected, rasterized, or resampled to 3 arc-second resolution, and matched to each rasterized buffered study area. Rasterized categorical variables were then converted into binary raster covariates, and subsequently utilised to generate continuous ‘*distance to*’ raster covariates (Table 2).

Bespoke measures were taken to prepare the land cover covariate variables. Similarly, to the aforementioned raster datasets representing categorical variables, the obtained C3S Global Land Cover data was projected, resampled to 3 arc-second resolution, and matched to the rasterized study area. The recoded global landcover dataset was then reclassified according to Sorichetta *et al*.^28^. Each land cover class was extracted and converted to a binary variable indicating presence/absence of the specified land cover class. Binary raster covariates were extracted to 100 km buffered study area raster datasets, and subsequently used to produce continuous ‘*distance to*’ raster covariates for each study area (Table 2). When a certain land cover class was completely absent, the covariate was disregarded for that specific country during RF model fitting and estimation, as on balance, the absence of the land cover type would not influence population distribution. The final land cover class (210) representing water and permanent ice cover distribution was disregarded in RF model fitting. Instead, the ESA’s waterbodies dataset was implemented as the ‘*distance to*’ water covariate variable, due to the improved spatial resolution it offers compared to the C3S Global Land Cover dataset (Table 2). Moreover, where available, waterbodies from the administrative unit boundary shapefiles were identified, rasterized, and incorporated into the waterbodies dataset, which was then processed using similar steps to the other raster datasets representing categorical variables, producing ‘*distance to*’ water covariate data for each country.

The distance to settlement covariate was prepared in the same way, generating a binary layer of building presence/absence from the WSF3D building area datasets; subsequently, ‘distance to’ settlement covariates were generated. The WSF3D building height data was prepared using a slightly differing methodology to the other continuous covariates; the settlement height data was extracted to the binary layer of WSF3D building presence/absence, instead of the official administrative boundaries. This measure ensures uniformity of building footprint delineation across all settlement covariates. ‘*No Data*’ grid cells in the building height layer overlapping the rasterized study area extent were filled with 0 values of the nearest neighbouring pixels. These building area and height datasets were multiplied using raster calculator to generate a building volume covariate.

**Fig. 1:**
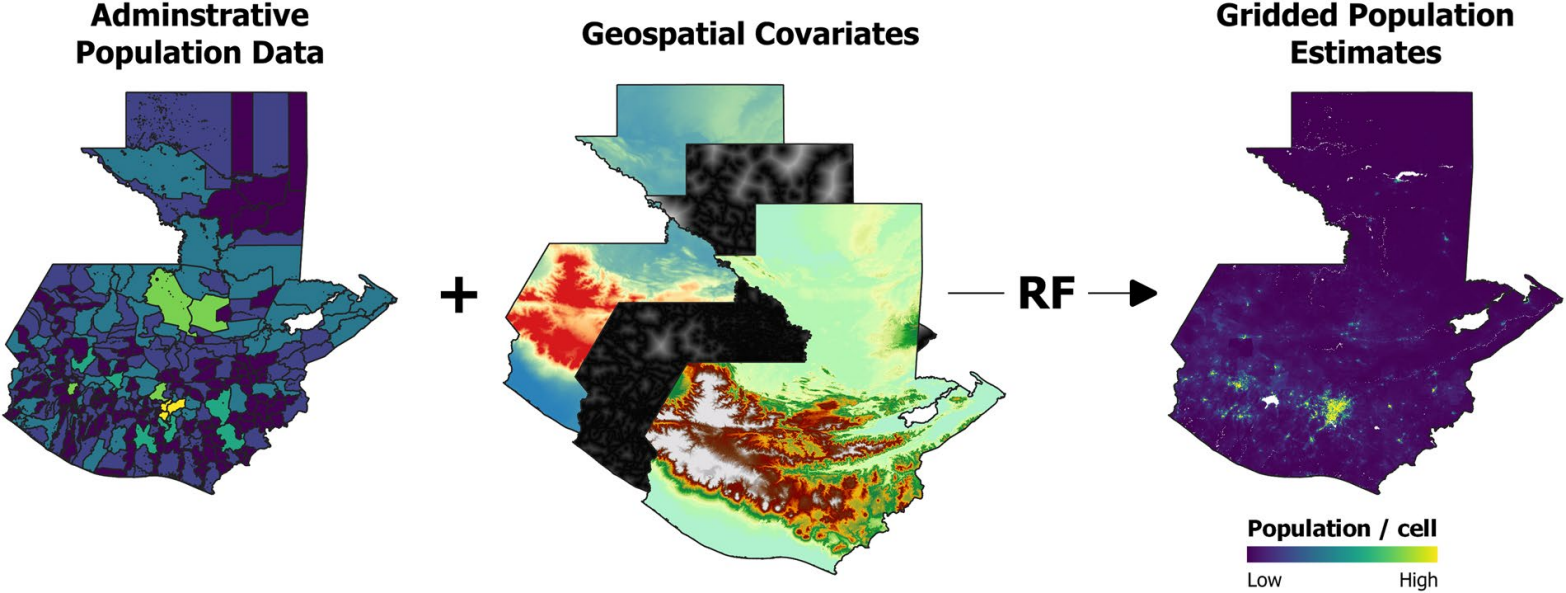
Schematic overview of the approach to generate gridded population estimates using the random forest (RF) model. For illustrative purposes, only a reduced set of considered covariates are shown here.

Moreover, a bespoke road classification system was established and applied to the raw OpenStreetMap data, using the ‘*fclass*’ field. This classification system comprised three distinct classes: (i) Pedestrian access, (ii) Motor-vehicle access, and (iii) residential roads (Table 3). The application of this custom classification system aims to aid the improvement of population estimations, by providing enhanced covariate detail. Furthermore, vector point data representing road intersections was generated for each road class using ArcGIS’s Intersect tool. These vector data were used to generate ‘distance to’ covariates for road and road intersection features for all countries, matching the corresponding spatial resolution of 3 arc seconds Figure 1.

**Table 3:** Reclassification of OSM roads and road intersection data.

bus_guideway	bus_stop	motor vehicle access
busway	construction	
emergency_access	emergency_access_point	
emergency_bay	escape	
mini_roundabout	motorway	
motorway_junction	motorway_link	
planned	primary	
primary_link	proposed	
raceway	rest_area	
road	secondary	
secondary_link	service	
services	tertiary	
tertiary_link	track	
track_grade1	track_grade2	
track_grade3	track_grade4	
track_grade5	trunk	
trunk_link		
bridleway	corridor	pedestrian access
crossing	cycleway	
elevator	footway	
living_street	path	
pedestrian	platform	
steps		
residential		residential
abandoned	disused	omitted
dummy	no	
unclassified	unknown	
yes		

**Fig. 2:**
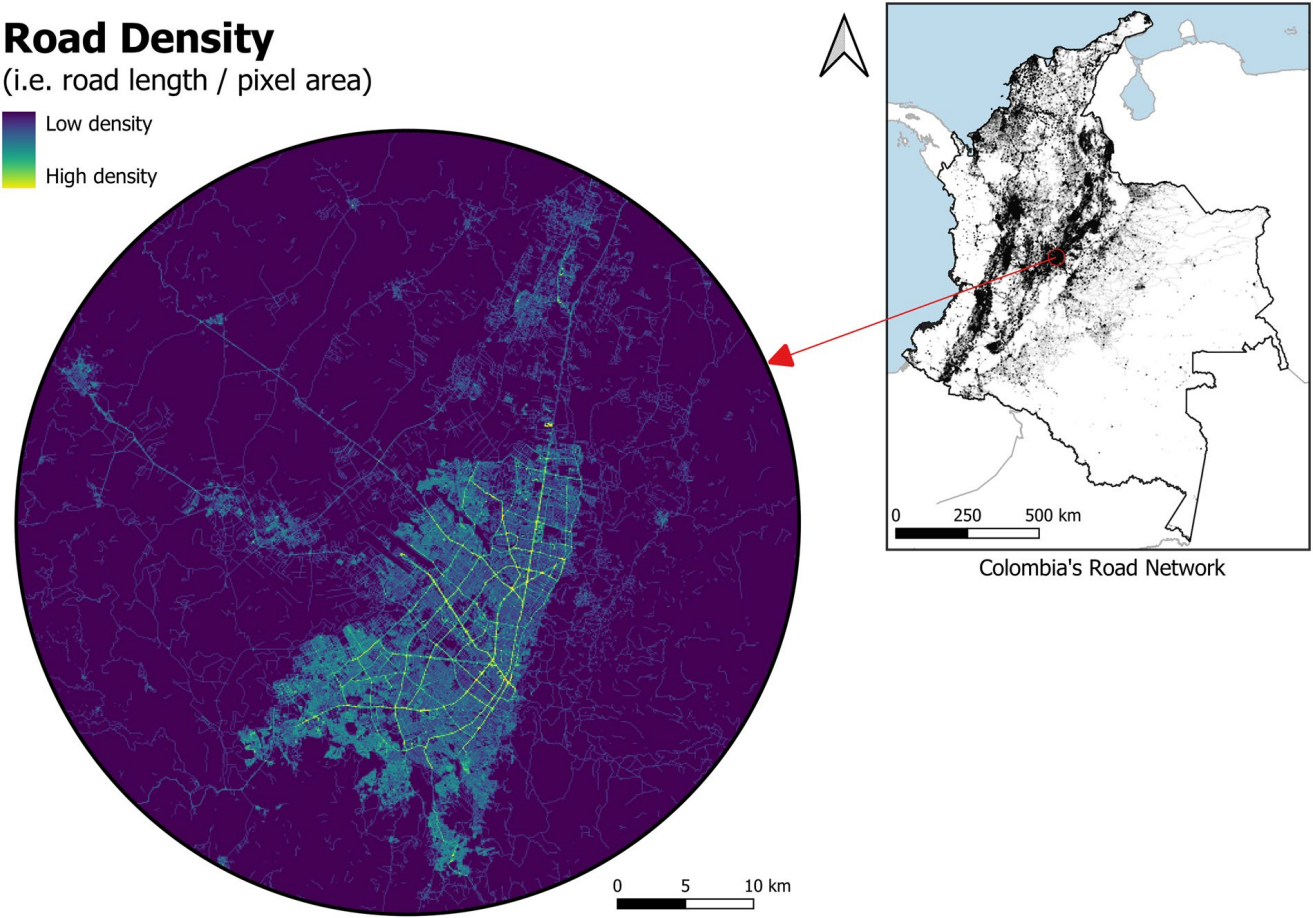
Road density in Bogotá, Colombia (3 arc second resolution).

Vector road data were also used to produce road density covariate of corresponding spatial resolution raster. Road density is defined as the ratio of the length of the roads in the pixel to the land area of the pixel. Therefore, vector data of roads was intersected with a raster grid at a resolution of 3 arc seconds (approximately 100 m at the equator) to ensure that each pixel has exact information for the roads within this pixel. Figure 2 shows the example of road density in Colombia.

**Fig. 3:**
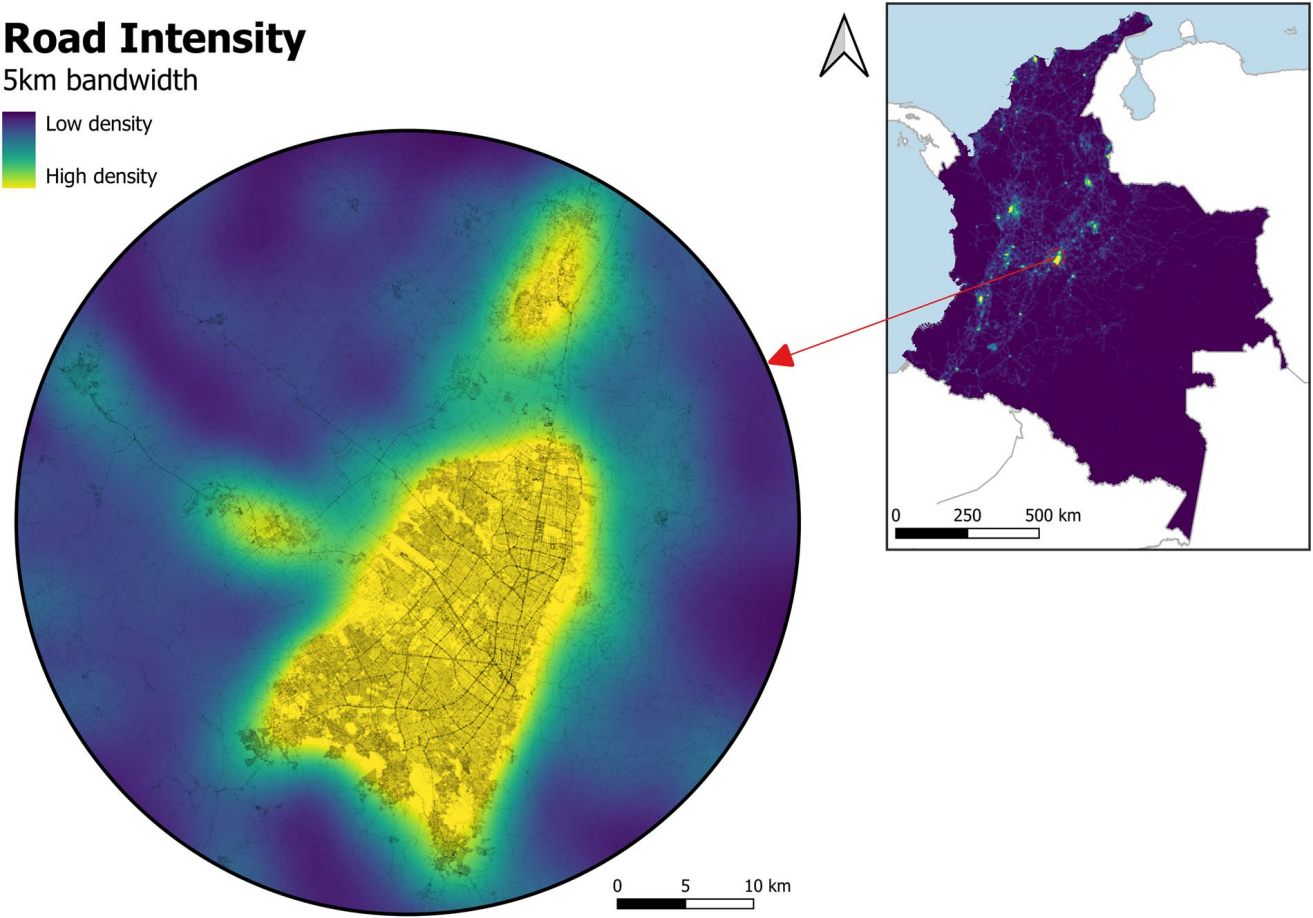
Road intensity (5 km bandwidth) in Bogotá, Colombia (3 arc second resolution).

Furthermore, in order to estimate the road density within a grid cell/pixel neighbourhood, a non-parametric ‘kernel’ method was used. Using the kernel approximation, one can achieve a smoother density estimate, compared to that of a coarser distribution. Therefore, to investigate the effect of road density at different spatial scales, 4 bandwidths (500 m, 1000 m, 2000 m and 5000 m) were used for the kernel density estimations. Road intensity was calculated using Epanechnikov kernel function^75^. Figure 3 shows the example of road intensity in Colombia.

### Random forest modelling scenarios

A set of modelling scenarios were devised to define the importance of covariate parameters for model fitting and prediction, as well as to enable the undertaking of a technical validation (Table 4). Specifically, the utility of WSF3D datasets when integrated into the RF modelling approach were to be assessed to assist the identification of the best final dataset for each country. Additionally, a simple areal-weighting (SAW) approach was generated as a comparison to assess the accuracy of RF-based dasymetric population modelling. These scenarios are detailed below (Table 4).

**Table 4:** Descriptions of modelled population distribution scenarios.

RF Scenario No.	Scenario Name	Scenario Description
1	Base RF	‘Finest-available’ units, fitted with all base covariates (i.e. all covariates excluding WSF3D-derived data).
2	Base RF & BA	‘Levelled-up’ units, fitted with all base covariates including building area.
3	Base RF & BH	‘Levelled-up’ units, fitted with all base covariates including building height.
4	Base RF & BABH	‘Levelled-up’ units, fitted with all base covariates including building area and building height.
5	Base RF & BABHBV	‘Levelled-up’ units, fitted with all base covariates including building area, building height, and building volume.
	SAW/Equal	‘Levelled-up’ units, simple areal-weighting approach.

‘Finest-available’ units refers to administrative units at their lowest available level, whilst ‘Levelled-up’ units refers to bespoke administrative units, generated via aggregating contiguous administrative units at the ‘finest-available’ level.

## Data Records

The high-resolution gridded population datasets detailed in this paper referring to the 40 countries listed in Table 1, are publicly and freely available through the WorldPop Data Repository^76^. The datasets can be downloaded as WinRAR Zip archives (win-rar.com) containing the population distribution datasets of the associated country for each of the five different RF modelling scenarios (Table 5).

**Table 5:** Name, description, and format of all files contained in each WinRAR zip archive related to the 40 countries listed in Table 1.

Name	Description	Format
ppp_ISO_RF1_v1.tif	Estimated people per grid cell for random forest covariate modelling scenario 1 (3 arc seconds)	GeoTIFF
ppp_ISO_RF2_v1.tif	Estimated people per grid cell for random forest covariate modelling scenario 2 (3 arc seconds)	GeoTIFF
ppp_ISO_RF3_v1.tif	Estimated people per grid cell for random forest covariate modelling scenario 3 (3 arc seconds)	GeoTIFF
ppp_ISO_RF4_v1.tif	Estimated people per grid cell for random forest covariate modelling scenario 4 (3 arc seconds)	GeoTIFF
ppp_ISO_RF5_v1.tif	Estimated people per grid cell for random forest covariate modelling scenario 5 (3 arc seconds)	GeoTIFF

In first column, ISO and RF represents the country ISO-3 code and RF covariate modelling scenario number respectively. All available data records were modelled using ‘finest-available’ units.

## Technical Validation

A technical validation framework was incorporated into the RF-modelling package, to ensure that the modelled population distribution outputs for each country and its administrative units were matching their population data input counterparts. However, as demonstrated by prior studies a ‘true-validation’ of gridded population datasets remains a significantly complex challenge due to the lack of high-resolution ground-truth data (i.e. population counts at the pixel level) required for an independent accuracy assessment of large-scale population models^73^.

**Fig. 4:**
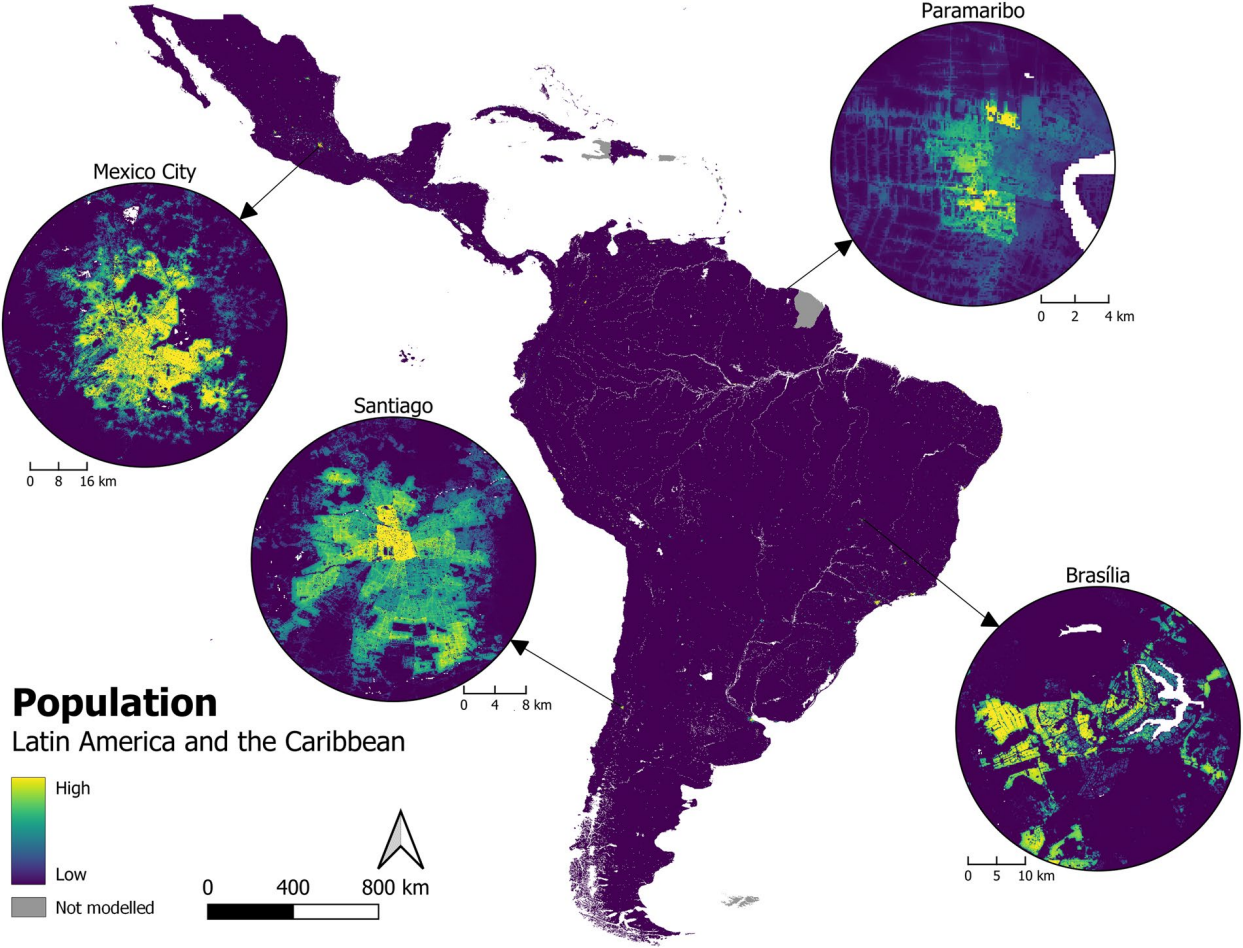
Estimated people per grid cell for 40 countries in Latin America and the Caribbean. Fitted using all base covariates including built area layers (for specific years see Table 1).

**Fig. 5:**
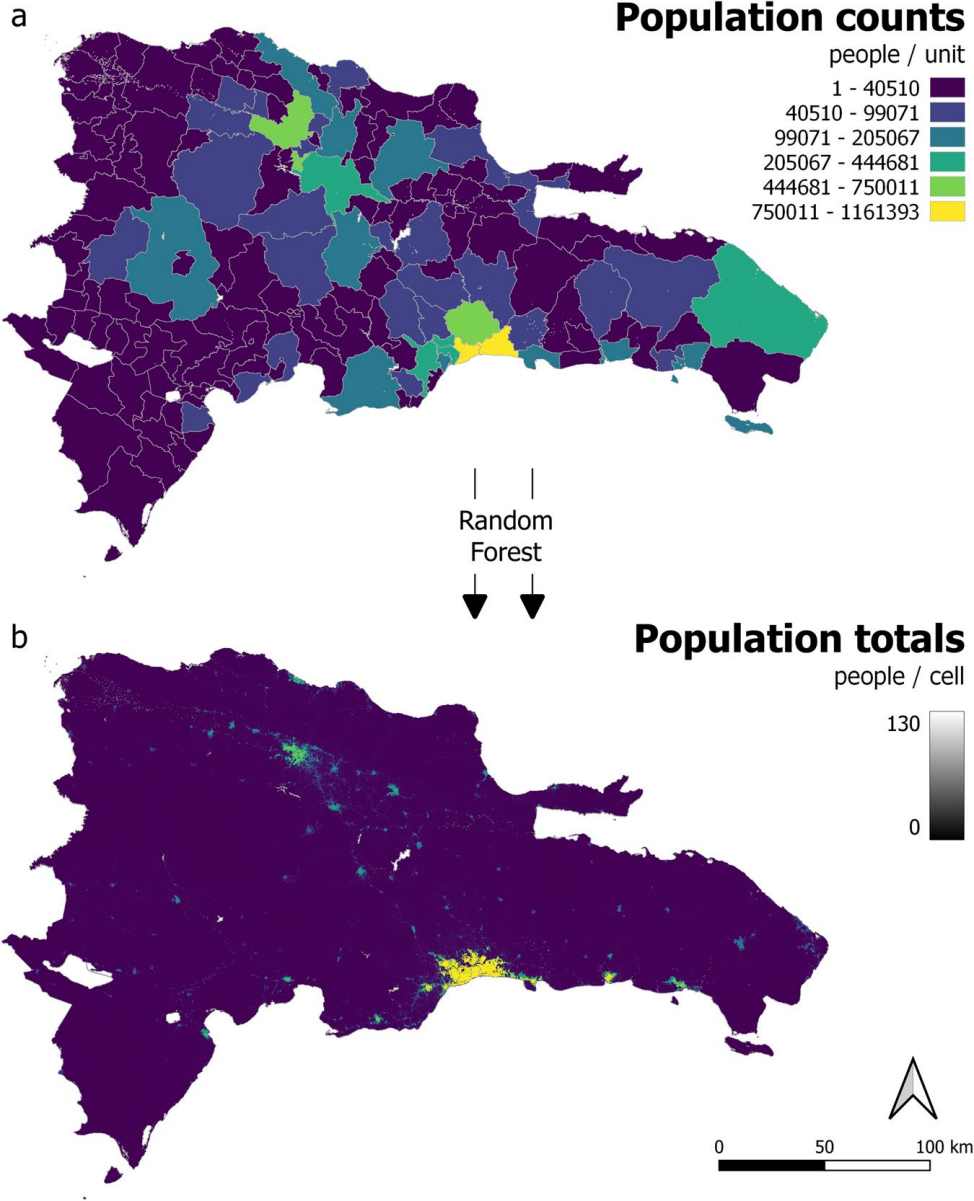
Population distribution in Dominican Republic, 2020. (**a**) input count data at ‘finest-available’ administrative unit level, (**b**) modelling outputs following random forest fitting at 3 arc second resolution (approximately 100 m at the equator), RF model fitted according to Scenario 6 (Table 4).

Firstly, the technical validation framework calculates zonal sums in the RF-output population distribution Figure 4, and checks the total population per administrative unit for the RF-output distribution against the input population data (e.g. Figure 5). This ensures that the population total within each administrative unit for RF-model outputs, matches the population total within corresponding administrative units for population inputs data prior to the RF-modelling.

**Fig. 6:**
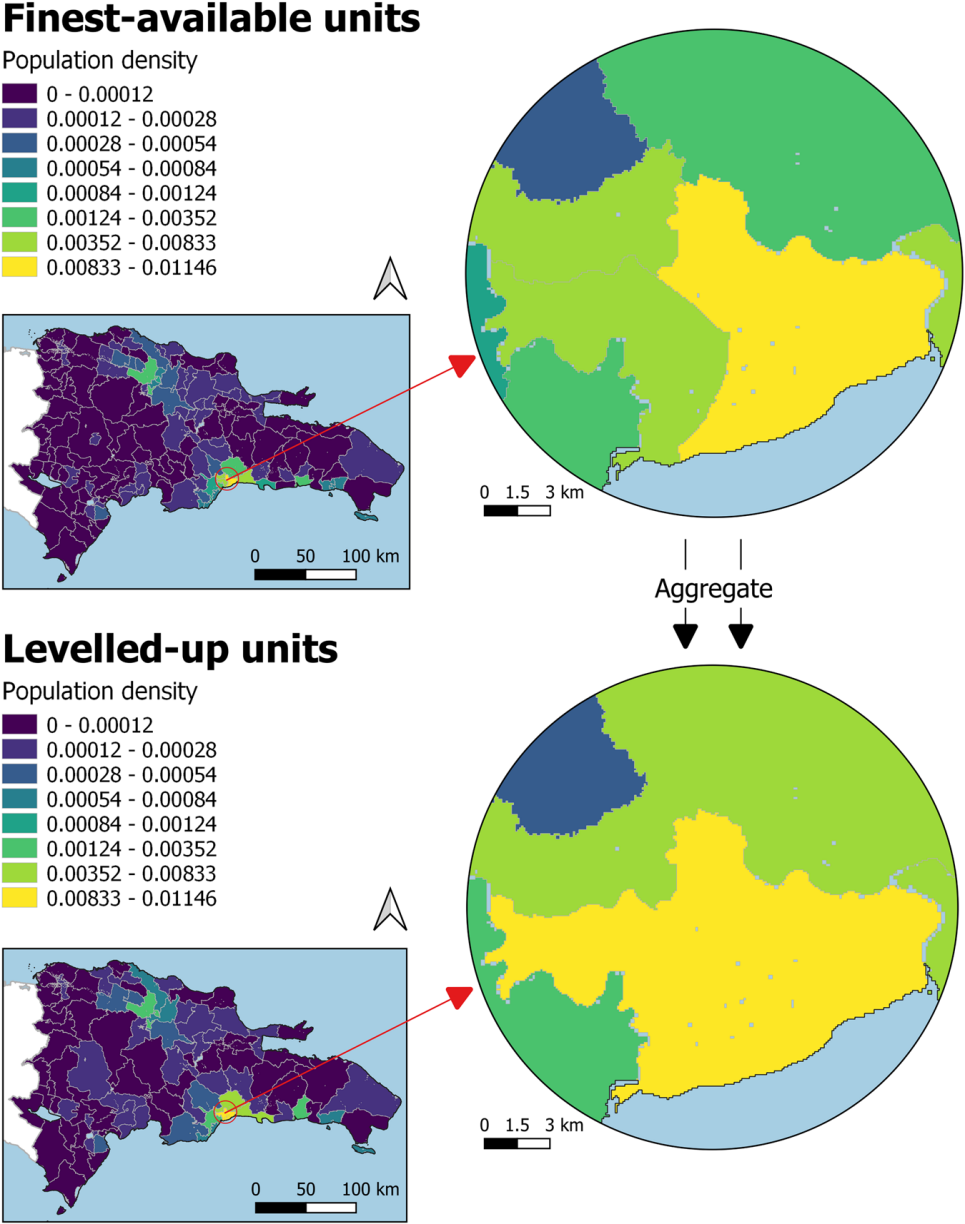
Comparison of ‘finest-available’, and the ‘levelled-up’ administrative units for Dominican Republic, 2020. ‘Finest-available’ units generated via aggregation of contiguous units.

In addition to this primary technical check, the existing research in the field of large-scale population modelling has utilised a validation method that quantifies the internal accuracy of population distribution method, in terms of “how well and plausibly populations are distributed”^77^. This framework performs a selection of statistical analyses using the differences between population counts extracted from distributions modelled using a coarser level of administrative units (‘*levelled-up*’), and the population counts of the finest available administrative units (‘*finest-available*’), here, the official population count data^69,73,78^. To generate this coarser administrative level, population counts were aggregated for each country by merging together pairs of contiguous administrative units characterised by similar population density values; this method was chosen with the aim to merge pairs of low population density units together and pairs of high population density units together (Figure 6).

A subset of countries (Table 6), located in different parts of the LAC region, were selected to assess the increased accuracy of the RF-based dasymetric mapping approach with respect to a SAW approach assuming that the population of each administrative unit is evenly distributed within it; this subset is defined as countries with sufficient administrative units following aggregation (minimum of 25) to fit the RF model. Although it is possible to fit the RF model for a given country with fewer than 25 administrative units by pairing it with an additional country with similar characteristics, it was deemed that the influence of the additional, finer-resolution country object would distort the validation of the modelling approach. Therefore, these countries were omitted from the subset.

**Table 6:** Prediction accuracy of the RF model used to generate dasymetric weighting layers compared to the ‘*levelled-up’* mapping approach.

ISO	Finest-Available			Levelled-Up		
	No. of units	OOB error	% of variance explained	No. of units	OOB error	% of variance explained
ABW	55	0.61	57.9	29	1.29	48.5
ARG	525	0.18	97.2	282	0.19	97.0
BRA	5,570	0.08	95.9	3020	0.08	96.1
CHL	246	0.27	96.0	190	0.38	94.1
COL	1,122	0.14	93.8	594	0.16	92.9
CRI	478	0.14	96.7	252	0.14	96.7
CUB	168	0.24	86.7	91	0.29	84.3
CUW	65	0.68	78.2	35	0.60	78.4
DOM	155	0.15	90.0	83	0.19	87.3
GTM	340	0.20	82.4	183	0.22	80.9
HND	298	0.11	83.6	160	0.11	84.0
MEX	2,457	0.15	94.0	1317	0.12	95.7
NIC	153	0.17	89.0	81	0.24	84.9
PER	1,873	0.20	94.4	995	0.19	94.6
PRY	250	0.16	95.1	135	0.21	93.6
SLV	262	0.21	81.0	142	0.18	83.8
SUR	62	0.63	94.1	33	0.72	93.2
Grouped Islands*	216	0.44	88.4	—	—	—

The OOB error and percentage variance explained are provided for 17 countries in addition to the ‘*Grouped Islands’* set of countries. A mapping approach using ‘*levelled-up*’ administrative units for these ‘*Grouped Islands*’ was not possible; the aggregating approach is not yet robust enough to handle the correct aggregation of contiguous administrative units for a collection of small, distinct islands.

### Model validation

The OOB error estimate (Table 6) is calculated during RF model fitting, and serves a robust and unbiased metric of the model’s internal prediction accuracy^34^. However, the OOB error estimate cannot be understood as the prediction error at the grid cell level, given that the RF model is fitted at the finest-available administrative level but predicts at the grid cell level. Furthermore, it should not be considered as the prediction error at the administrative unit level, via totalling of all final grid cell values within each administrative unit, and comparing it to the observed population count of the equivalent administrative unit. Nevertheless, it is expected that a higher accuracy of predicted values at the administrative level, should be associated with higher accuracy of the final gridded population distribution datasets^33^.

Between ‘*finest-available*’ and ‘*levelled-up*’ modelling scenarios, the OOB error increased and the percentage of variance explained decreased for 10 countries amongst the subset: ABW, ARG, CHL, COL, CUB, DOM, GTM, NIC, PRY and SUR (Table 6). The most significant difference is noted for ABW in which the OOB error more than doubled, whilst percentage of variance explained reduced by almost 10% (Table 6). However, the degree of differences in OOB error and percentage of variance explained were much less significant for the remaining countries within the subset (Table 6). There are some examples of countries in which the ‘levelled-up’ scenario exhibited reduced OOB error values and higher percentage of variance explained, compared to the ‘finest-available’ modelling output; most notable amongst these are MEX and SLV (Table 6). The OOB error value for both MEX and SLV decreased by 0.03, whilst the percentage of variance explained increased by 1.7% and 2.8%, respectively (Table 6).

### WSF3D quantitative assessment

For each country within this subset, a selection of spatial error metrics were identified and calculated to assess the accuracy and reported differences between the actual and the estimated values for each country’s ‘*finest-available*’ administrative unit; in this case the actual values are obtained from the input population count data at the *‘finest-available*’ administrative unit level, whilst the estimated values are derived from Zonal Statistics sum calculations of each resultant RF modelling scenario output (Table 4) at the same ‘*finest-available*’ administrative level. For each country (Table 6) and each modelled RF-scenario (Table 4), the following error metrics are derived in Table 7.

**Table 7:** Descriptive metrics for accuracy assessment at the validation unit level for modelling scenarios (Table 4).

Metric	Description
$MA{E}_{i}=\frac{{\sum }_{i\in L1=1}^{n}| po{p}_{a}-po{p}_{e}| }{n}$ (1)	MAE is the mean absolute error at each level of analysis (i), calculated as the mean of the total error, i.e. the sum of absolute differences between the actual (pop_a_) and the estimated population (pop_e_) at each validation unit.
$MAP{E}_{i}=\frac{MA{E}_{i}}{Av.Pop}$ (2)	MAPE is the mean absolute percentage error at each level of analysis (i), calculated as the MAE_i_ divided by the average population of each country.
$RMS{E}_{i}=\sqrt{\frac{{\sum }_{i\in L1=1}^{n}{(| po{p}_{a}-po{p}_{e}| )}^{2}}{n}}$ (3)	RMSE is the root mean square error at each level of analysis (i), calculated as the square root of the total square error, i.e. the average of the sum of squared errors (pop_a_ – pop_e_) at each validation unit.
$RMSP{E}_{i}=\sqrt{\frac{{\sum }_{i\in L1=1}^{n}{(\frac{| po{p}_{a}-po{p}_{e}| }{po{p}_{a}})}^{2}}{n}}$ (4)	RMSPE is the root mean square percentage error at each level of analysis (i), calculated as the square root of the mean of the sum of total errors divided by actual population squared, at each validation unit.

For metrics capturing ‘*percentage error*’, the respective measures are multiplied by 100 to convert them to percentages.

For each country, four accuracy metrics were used to assess how well each RF modelling scenario distributed the population. Both the Mean Absolute Error (MAE) and Root Mean Square Error (RMSE) measure the absolute differences between the actual (pop_a_) and estimated population (pop_e_) counts of the L1-base units^73,79^. However, MAE is known to be more robust to outliers^80^, since RMSE penalises significant errors by squaring differences, compared to MAE which weights each error equally^73^. Conversely, the Mean Absolute Percentage Error (MAPE)  is the MAE adjusted to each level of analysis, calculated as MAE divided by the average population of each country^78^. Similarly, the RMSE is also expressed as a percentage of the mean population size of the administrative unit level via the root mean square percentage error (RMSPE). These metrics enable comparison across countries by omitting the bias caused by different population totals and number of administrative units; furthermore, ‘*percentage error’* metrics help to determine if errors generated by different modelling layers are similar and systematic, or if different behaviours are observable across countries^73^.

**Fig. 7:**
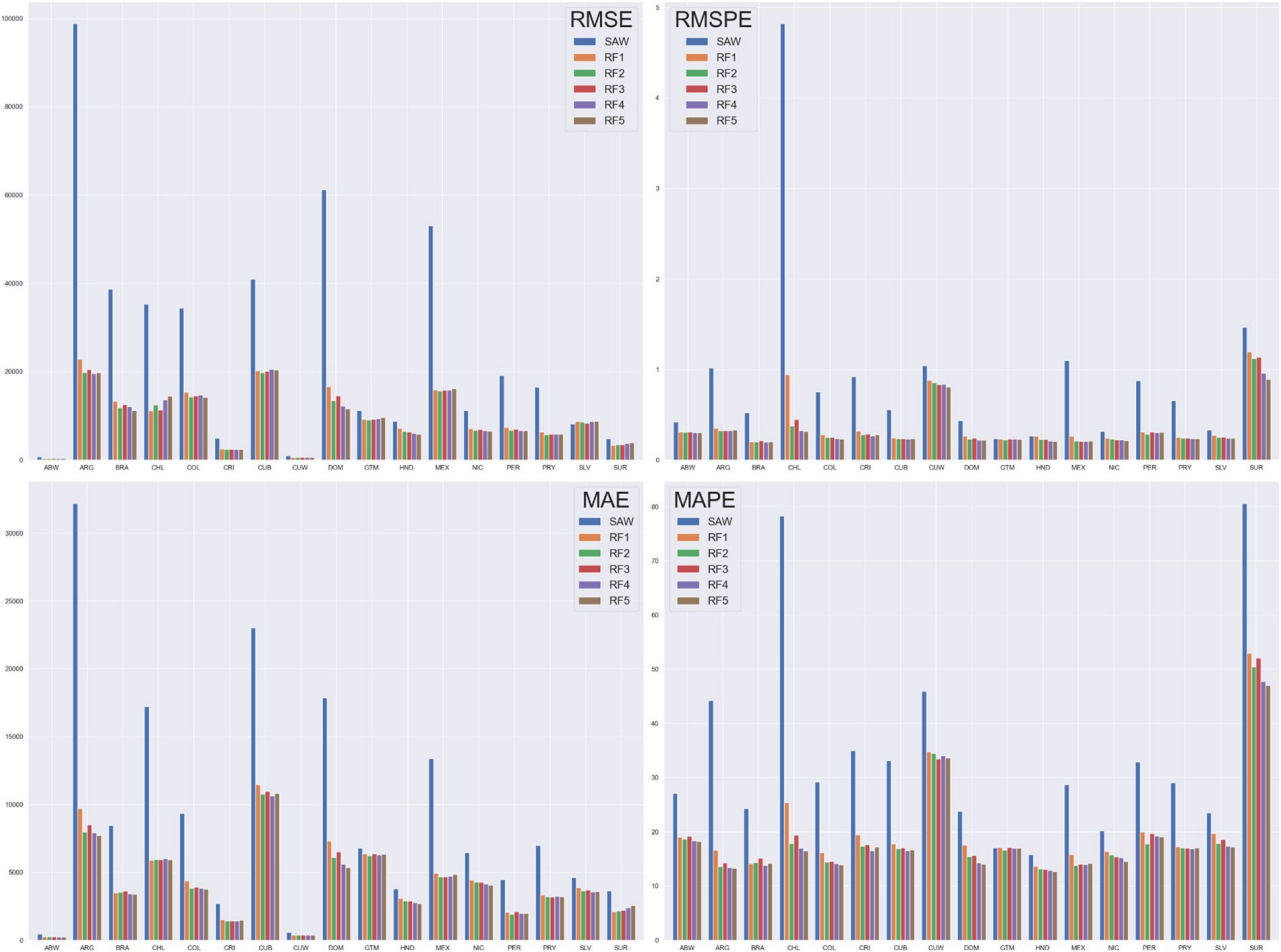
Accuracy assessment results for modelling population density of all scenarios (Table 4) for each country (ISO-3).

Results, summarised in Figure 7, indicate that the high-resolution gridded population datasets produced under this project’s framework outperform their corresponding SAW-based outputs across almost all cross-sections of metrics, countries, and modelling scenarios. The first exception to this finding is El Salvador (SLV), in which the calculated RMSE value increases from 8159 to a maximum of 8779 between the SAW and *Scenario 6* (Table 4) modelling approaches, respectively (Figure 7). The second exception is Guatemala (GTM), in which the calculated MAPE value of 17.05% for the SAW modelling approach is lower than *Scenarios 1* and *3* (Table 4); nevertheless the remaining modelling scenarios for Guatemala (*Scenarios 2, 4*, and 5) are an improvement on the SAW-based outputs according to calculated MAPE values of 16.64, 17.00, and 16.96, respectively (Figure 7).

Beyond these exceptions, according to the calculated accuracy assessment metrics (Figure 7), *Scenario 6* (Table 4) is the best performing modelling method for 31 of the 68 country-accuracy metric combinations. Moreover, *Scenarios 3* and 5 are the next best performing modelling methods, registering the best accuracy metric result for 14 and 13 of the 68 country-accuracy metric combinations, respectively; as discussed above, the SAW-based modelling approach was found to be the best performing modelling scenario in only one case (SLV-MAE). These findings highlight a number of concepts, including (i) the importance of the building area covariate to RF-model fitting, (ii) the value of integrating all building covariates to RF-model fitting, and (iii) the increased accuracy of the RF dasymetric disaggregation approach compared to a SAW-based disaggregation.

## Usage Notes

In particular, the presented gridded datasets provide improved spatial detail of the residential population distribution at sub-administrative unit level comparatively to most publicly-available (i) administrative unit-level official and non-official estimates or projections, which implicitly rely on the assumption that the population is homogeneously distributed within each units, and (ii) gridded population datasets, which are based on non-official estimates or projections. This is achieved via the disaggregation of the most recent and finest administrative unit-level official population projections, produced by 40 NSOs and processed with support by the UNFPA Regional Office for Latin America and the Caribbean, UNFPA’s Population and Development Branch and the Information Management Branch of the UN Office for the Coordination of Humanitarian Affairs (OCHA).

Furthermore, these gridded population distribution datasets represent a consistent and comparable format, as well as a scalable framework, providing flexibility in (i) summarisation to any spatial area of interest (e.g., areas impacted by natural and/or man-made hazards which may not correspond to predefined artificial administrative boundaries), and (ii) analysis and data integration (e.g., GIS and remote sensing data, such as locations of healthcare facilities and CO_2_ emissions, respectively). Thereby, they can be effectively considered for  planning and supporting interventions and applications (e.g., planning for elections, assessing exposure to natural hazards, and measuring demand for services), measuring progress (e.g., measuring and monitoring the SDGs and their indicators), and performing analyses (e.g., predicting response variables intrinsically dependent on the population distribution, and modelling epidemic spreads).

However, it is important to note that there are also a number of limitations, caveats, and assumptions inherent in the modelling approach used to produce the gridded population datasets, that should be considered before using them. For consistency, all datasets were produced using a fixed number of ancillary covariates available for all countries, and thus only a limited number of factors, potentially related to population presence and densities in each country, have been considered overall. For this reason, which represents a trade-off in the production of generalizable models, the accuracy of the gridded population datasets for some of the countries could be improved by considering additional, locally-specific factors that could help to increase the percentage of variance explained by the corresponding RF model.

Other limitations are represented by (i) the fact that the spatial detail of the administrative unit-level population projections was not the same for all countries (refer to the “Unit level” column in Table 1), with the use of smaller administrative units for a given countries translating into higher accuracy of the corresponding gridded population dataset, and (ii) the fact that, because of the lack of enough administrative units to fit a country specific RF model, the gridded population datasets for a number of countries and islands have been produced using RF models referring to another country or a set of countries, and “Grouped Islands”, respectively (refer to the “Modelled with” column in Table 1)^38^.

Additionally, it may be worth to reflect on the fact that the official administrative unit-level population census-based figures and projections, used as inputs to the RF model, may or may not have captured effects of potential rapid onset events responsible for abrupt fluctuations of population numbers at the administrative unit level (e.g., forced displacements due to natural disasters). Similarly, the gridded population datasets produced using them do not account for seasonal or intra-annual population mobility between administrative units.

Upon aggregation, gridded population datasets constructed using this disaggregation methodology are proved to be more accurate at representing human population distribution compared to those produced using an equal-area approach^28,33^. The reliability of the data product is unknown at the grid-cell level, therefore it is recommended that population datasets be aggregated before use instead of at the grid-cell level^77^. Furthermore, it is important to highlight that gridded population datasets give end-users the flexibility to aggregate population according to different boundaries and/or areas (i.e. boundaries and/or areas that do not align with the administrative unit boundaries of the input population data).

Furthermore, it is critical to consider that these gridded population datasets represent modelling outputs generated using a number of ancillary covariates and thus, to avoid circularity, they should not be used to make predictions about, or explore relationships with, any of the factors included in the model (e.g., correlating population distribution with settlement distribution). If there is such need, ideally, the modelling process should be re-run using the publicly and freely available WorldPop-RF code (https://github.com/wpgp/popRF) with the covariate of interest being removed to avoid issues relating to endogeneity.

Finally, it is also important to note that most of the considered ancillary covariates are derived from modelling outputs themselves, and thus they have a degree of uncertainty that carries over into the gridded population datasets.

## Data Availability

The WorldPop-RF code, used to produce these high-resolution gridded population datasets, is publicly and freely available via: https://github.com/wpgp/popRF.
